# Extensor retinaculum excision does not affect wrist tendon forces: a cadaveric simulator study

**DOI:** 10.1177/1753193420928781

**Published:** 2020-06-06

**Authors:** Darshan S. Shah, Maxim D. Horwitz, Angela E. Kedgley

**Affiliations:** 1 Department of Bioengineering, Imperial College London, London, UK; 2 Department of Hand Surgery, Chelsea and Westminster Hospital, London, UK

Dear Editor,

The extensor retinaculum prevents wrist and finger tendons from bowstringing during wrist extension, thereby preserving their moment arms (Garland et al., 2018). However, it is often compromised during trauma or elective wrist surgery; moreover, the retinaculum is harvested as an autograft in certain reconstructive procedures of the digits. The aim of this study was to quantify alterations in wrist tendon forces following extensor retinaculum excision and eventual surgical repair, using a physiological wrist simulator to replicate active wrist motions in vitro.

Nine fresh-frozen cadaveric specimens, thawed at room temperature for 12 hours before testing, were prepared for mounting on a validated physiological wrist simulator (Shah et al., 2017). Six muscles were selected – flexor carpi radialis (FCR), flexor carpi ulnaris, extensor carpi radialis longus, extensor carpi radialis brevis (ECRB), extensor carpi ulnaris and abductor pollicis longus – as these cross the wrist joint and insert at the base of the metacarpals, thereby primarily contributing to wrist motions. The tendons were dissected up to their musculotendinous junctions in order to permit free mobilization and gliding during testing. Electromechanical actuators applied tensile loads via steel cables affixed to the six tendons to recreate motion at the wrist. Joint kinematics were obtained using an eight-camera optical motion capture system (Qualisys, Gothenburg, Sweden) to track clusters of retroreflective markers fixed to the third metacarpal and radius. Tendon forces were monitored by load cells connected in series with the actuators. The respective position and force feedbacks were combined within a control strategy, which computed the distribution of forces across the wrist muscles in real time to accurately replicate active wrist motions (Shah et al., 2017).

Multiple cyclic motions of flexion–extension (FE) (50° flexion to 30° extension) and radioulnar deviation (RUD) (15° ulnar to 15° radial) were simulated in specimens with an intact extensor retinaculum. This was followed by a dorsal release of the retinaculum in a Z-plasty fashion, completely opening the second, third, fourth and fifth compartments. Each specimen was moved through the same cyclic motions of FE and RUD. The retinaculum was then approximated and repaired, the skin sutured and the cyclic motions again repeated. Tendon bowstringing was qualitatively assessed using videography. Muscle forces were evaluated as a function of joint kinematics and compared across the intact, resected and reconstructed cases at every 10° of FE and 5° of RUD using the Friedman test (*p* < 0.05), followed by Wilcoxon signed rank tests with Bonferroni adjustments for pairwise comparisons (*p* < 0.018). Differences in peak muscle forces larger than 10% were considered clinically important, as previously shown by Shah et al. (2019).

**Figure 1.**
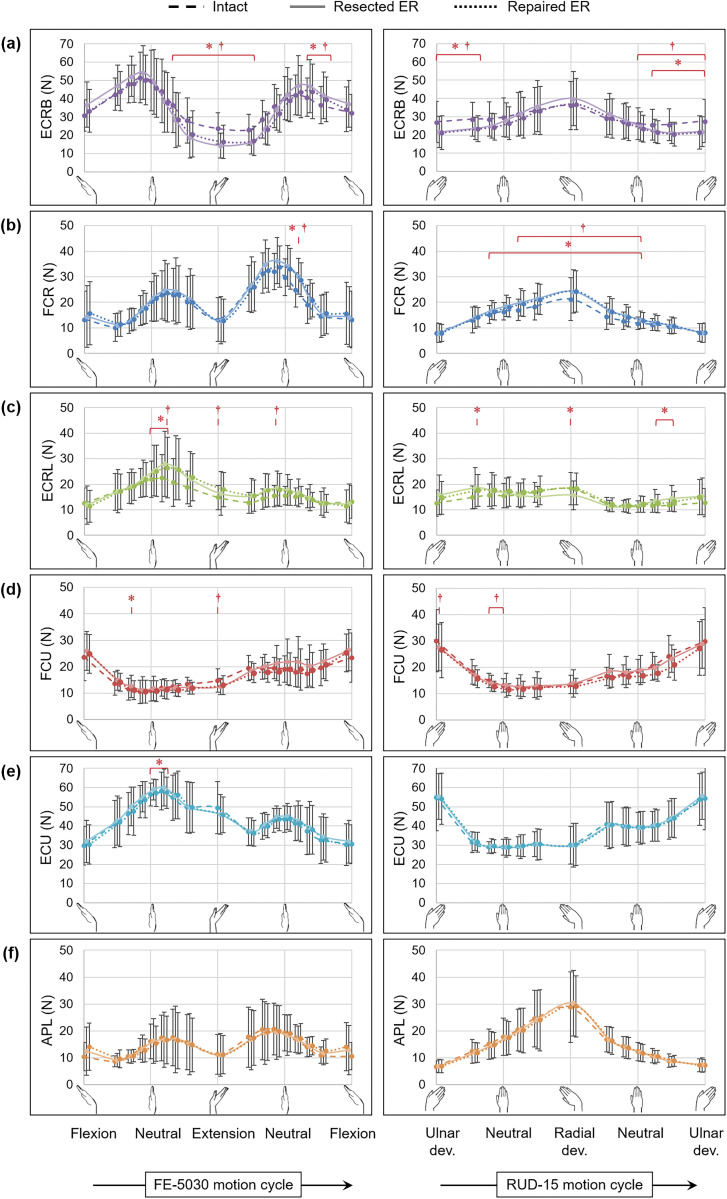
Muscle forces (Mean ± SD across nine specimens) of the (a) extensor carpi radialis brevis (ECRB), (b) flexor carpi radialis (FCR), (c) extensor carpi radialis longus (ECRL), (d) flexor carpi ulnaris (FCU), (e) extensor carpi ulnaris (ECU) and (f) abductor pollicis longus (APL) in flexion–extension (FE-5030) and radioulnar deviation (RUD-15) for the intact condition (dashed) and following the resection (solid) and repair (dotted) of the extensor retinaculum (ER). The asterisk (*) indicates statistically significant differences between the intact and the resected conditions, while the dagger (†) indicates statistically significant differences between the intact and the repaired conditions (significance: *p* < 0.018).

ECRB forces reduced by more than 30% in extension (*p* < 0.011) and 20% in ulnar deviation (*p* < 0.011) for both the resected and reconstructed conditions compared with the intact condition (Figure 1(a)); however, no differences were observed in mean ECRB force (*p* > 0.236). While the FCR force increased by 15% in radial deviation (*p* < 0.015) (Figure 1(b)), no differences were observed in the mean and peak forces of other muscles, among the intact, resected and reconstructed cases, throughout FE (*p* > 0.097) and RUD (*p* > 0.121).

Neither retinacular resection nor postsurgical repair resulted in clinically important alterations to wrist tendon forces, which supports the use of retinacular tissue as an autograft. The reduction in the ECRB force in extension and ulnar deviation (Figure 1(a)) was indicative of tendon bowstringing – wherein an increased tendon moment arm led to a reduction in tendon force required to achieve the same joint torque – as was also reported by Brody and Merrell (2015). Moreover, findings from the current study suggested that bowstringing did not have clinically significant effects on the distribution of wrist tendon forces across the ECRB and its synergists and/or antagonists.

No significant differences were observed in muscle forces between the resected retinaculum and repaired retinaculum conditions (Figure 1). Although retinacular repair is recommended to prevent bowstringing (Skillman and Belcher, 2003), the current methodology involves suturing the superficial transverse tissue alone, but not with the vertical septae, which compartmentalize tendons. The defunct vertical septae, following resection or eventual repair of the superficial transverse tissue, might have allowed tendons to bowstring away from the carpals during extension and across the various resected compartments during RUD. However, although reconstruction of individual retinacular compartments could theoretically aid in preventing bowstringing, thereby avoiding abnormal excursion of extensors, the presence of tendon bowstringing following transverse tissue repair does not seem to result in any clinically significant alterations in muscle forces when compared with the intact condition.

Limitations of our study include the qualitative visual assessment of tendon bowstringing, as opposed to quantitative measurements, and the consideration of only six tendons to replicate wrist motions in vitro. Extrinsic muscles of the fingers and the thumb will be included in future experiments, since they contribute to wrist torque and exhibit prominent bowstringing, such as in the case of the extensor digitorum communis (Brody and Merrell, 2015).

In conclusion, although retinaculum resection or repair might lead to tendon bowstringing, they do not result in clinically important alterations of wrist muscle forces.
